# A dual cohort analysis of parenting practices, attention‐deficit/hyperactivity disorder symptoms, anger, and emotion dysregulation in middle childhood: Findings from a UK and Zurich sample

**DOI:** 10.1002/jcv2.70059

**Published:** 2025-10-19

**Authors:** Evelyn Mary‐Ann Antony, Nadin Beckmann, Steve Higgins

**Affiliations:** ^1^ School of Education Durham University Durham UK

**Keywords:** anger, attention‐deficit/hyperactivity disorder symptoms, emotion dysregulation, parenting practices

## Abstract

**Background:**

In middle childhood (ages 6–12), children with attention‐deficit/hyperactivity disorder (ADHD) symptoms often experience emotion dysregulation (ED), anger, and social difficulties, including peer problems and maladaptive conflict resolution. Parenting plays a critical role in shaping emotional development; however, how parenting practices interact with ADHD symptoms to influence ED, anger, and social functioning remains underexplored, particularly across cross‐national contexts. This study examined these dynamics using longitudinal data from two population‐based cohorts in the UK and Zurich.

**Methods:**

Data was drawn from two longitudinal cohorts: the UK Millennium Cohort Study (MCS; *n* = 3147–30,135) and the Zurich Project on Social Development from Childhood to Adulthood (z‐proso; *n* = 1179–1360). In the MCS sample, we ran cross‐sectional moderation models to examine whether ADHD symptoms moderated the association between withdrawn/harsh parenting practices and emotion dysregulation, at ages 5 and 7, respectively **(H1)**. The MCS sample also examined whether ADHD symptoms at age 7 moderated the association between withdrawn/harsh parenting practices at age 5 and peer relationship problems at age 11, longitudinally **(H2)**. In the z‐proso sample, we ran cross‐sectional moderation models to examine whether ADHD symptoms moderated the association between negative/positive parenting and anger, at age 9 **(H3)**. The z‐proso sample additionally focused on whether anger at 9 mediates the association between age 7 negative/positive parenting and conflict coping strategies (aggressive/competent) at age 11, longitudinally **(H4)** Multiple linear regression, moderation, and mediation analyses were performed, adjusting for demographic covariates, such as gender, income (MCS), socio‐economic status (z‐proso), maternal education (MCS), ethnicity (MCS), and parents' migration background (z‐proso).

**Results:**

In the MCS, cross‐sectionally at age 7, a statistically significant negative interaction was observed, indicating that the association between withdrawn parenting and ED was stronger at lower levels of ADHD symptoms and weakened as ADHD symptoms increased, suggesting that parenting had a greater influence on emotional outcomes when ADHD symptoms were less pronounced. Longitudinally, ADHD symptoms at age 7 amplified the effects of withdrawn parenting at age 5 on peer problems at age 11, highlighting the compounding risk of elevated ADHD symptoms and early parenting challenges on later social functioning. In z‐proso, cross‐sectionally at age 9, ADHD symptoms did not moderate the effects of positive or negative parenting on anger. Longitudinally, anger at age 9 mediated the association between ADHD symptoms at age 7 and both aggressive and competent conflict coping at age 11, identifying emotional reactivity as a key developmental mechanism linking early ADHD symptoms to later behavioural adjustment.

**Conclusion:**

Findings across both cohorts showed that parenting practices and ADHD symptoms interact to influence emotion dysregulation, anger, and social difficulties in middle childhood. Anger emerged as a distinct emotional process, reinforcing its unique role in social challenges. The results highlight the need for culturally sensitive interventions that address both parenting and emotion regulation to improve outcomes for children with ADHD symptoms.

## Key Points

**What is Known?**

- Children with attention‐deficit/hyperactivity disorder (ADHD) symptoms often struggle with co‐occurring emotion dysregulation (ED) and anger, with these difficulties intensified by negative parenting practices.

**What is New?**

- This study provides novel cross‐national insights into how parenting practices and ADHD symptoms interact to influence ED and anger in both the UK and Zurich.

**What is Relevant?**

- The findings underscore the importance of addressing withdrawn and negative parenting practices and integrating tailored interventions to improve socio‐emotional outcomes.

## BACKGROUND

Attention‐deficit/hyperactivity disorder (ADHD) is a neurodevelopmental disorder marked by persistent inattention and/or hyperactivity‐impulsivity, affecting approximately 5%–7% of children, both globally and in the UK (NHS Digital, 2023). ADHD frequently disrupts children's academic, emotional, and social development, particularly in middle childhood (ages 6–12), a sensitive period for self‐regulatory and interpersonal skills (De Raeymaecker & Dhar, 2022; Mah & Ford‐Jones, 2012). A core and impairing feature of ADHD is emotion dysregulation (ED), which significantly increases the risk of comorbid disorders such as anxiety, depression, and conduct problems (Antony et al., 2022; Beauchaine & Cicchetti, 2019).

Within the developmental psychopathology framework, both individual traits (e.g., ADHD symptoms, anger reactivity) and environmental factors (e.g., parenting, peer relationships) interact to shape long‐term outcomes (Murray et al., 2022). Emotion dysregulation serves as a transdiagnostic mechanism and developmental pathway that links ADHD symptoms to later maladjustment, while anger, an intense, often poorly regulated emotion, can be considered a behavioural manifestation of ED (Vacher et al., 2020). Children with ADHD symptoms often show heightened anger responses and struggle to modulate them, which contributes to peer rejection and interpersonal conflict (Glenn et al., 2021).

Social difficulties are particularly pronounced in children with ADHD due to deficits in social cognition, impulse control, and conflict resolution (Barkley & Fischer, 2019; Lee et al., 2021). Anger and poor coping strategies further exacerbate these difficulties, creating a feedback loop of peer rejection and maladjustment (Willoughby et al., 2012). While anger may be viewed as a proxy for ED in many cases, it also warrants separate consideration due to its unique role in triggering reactive aggression and social disruption. Parenting practices critically shape these developmental pathways. Harsh or withdrawn discipline increases the likelihood of emotional distress, aggression, and peer conflict (Breaux et al., 2018), while positive parenting supports emotion regulation and prosocial skills. However, parenting is not uniform across contexts; cultural values influence discipline strategies, perceptions of ADHD symptoms, and emotional socialisation (Lansford, 2022). Cross‐national comparisons are therefore essential for understanding how parenting interacts with child characteristics to predict long‐term adjustment.

In this study, we draw on data from two population‐based longitudinal cohorts, the UK Millennium Cohort Study (MCS) and the Swiss‐based Zurich Project on Social Development from Childhood to Adulthood (z‐proso), to examine how ADHD symptoms, anger, parenting practices, emotion dysregulation symptoms, and social outcomes interact across middle childhood and cross‐national settings. Rather than combining samples, we use each dataset to address distinct but conceptually linked questions. Specifically, both studies test how ADHD symptoms influence or are influenced by parenting practices and emotional responses (anger, ED), and how these pathways predict social competence or difficulties (peer problems, conflict coping). This aligns with an integrated model of developmental risk, where early ADHD‐related vulnerabilities interact with family context to shape emotion regulation and social trajectories (Greenhoot & Dowsett, 2012; Sonuga‐Barke, 2013).

Importantly, while ED is a latent construct spanning emotion awareness, modulation, and expression, anger is conceptualised here as a discrete, observable component of ED that may have unique mediating effects on social functioning. Peer problems and conflict coping are treated as key social outcomes shaped by these emotional dynamics. Longitudinal designs are crucial to establish directionality and examine how early parenting and ADHD symptoms jointly influence emotional and social development over time (Murray et al., 2022). This is, to our knowledge, the first study to examine both cross‐sectional and longitudinal associations between ADHD symptoms, parenting, anger/ED, and social outcomes across two cross‐national contexts. The use of large, nationally representative samples enables testing developmental pathways in naturalistic settings and comparing mechanisms across cross‐national groups with different parenting norms and structural support.

### Hypotheses

We hypothesise the following:

- **H1 (MCS, cross‐sectional)**: ADHD symptoms moderate the association between parenting discipline practices (withdrawn/harsh) and ED symptoms at ages 5 and 7, respectively.
- **H2 (MCS, longitudinal)**: ADHD symptoms at age 7 moderate the relationship between discipline parenting (withdrawn/harsh) at age 5 and peer relationship problems at age 11.
- **H3 (z‐proso, cross‐sectional)**: ADHD symptoms moderate the association between parenting practices (negative/positive) and anger at age 9.
- **H4 (z‐proso, longitudinal)**: Anger at age 9 mediates the relationship between ADHD symptoms at age 7 and conflict coping strategies (aggressive/competent) at age 11.

**FIGURE 1:**
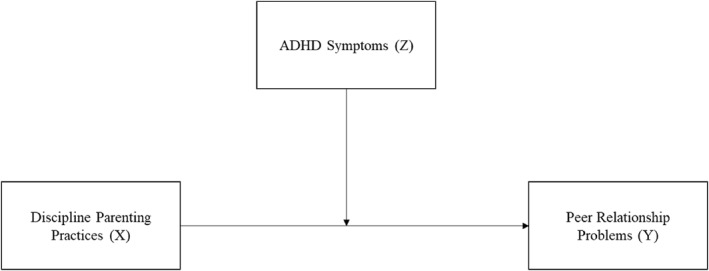
Example path moderation model with no covariates (MCS). MCS, Millennium Cohort Study.

**FIGURE 2:**
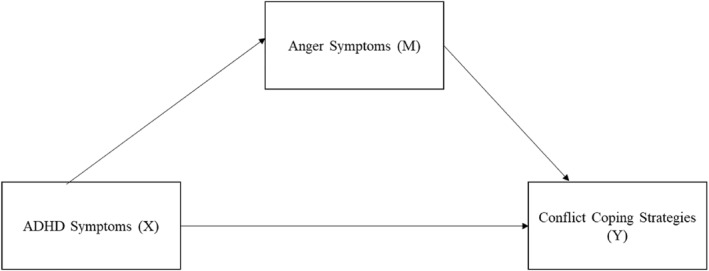
Example path mediation model with no covariates (z‐proso). z‐proso, Zurich Project on Social Development from Childhood to Adulthood.

Figures 1 and 2 depict the conceptual moderation and mediation models for the MCS and z‐proso data, respectively.

## METHOD

We used data from two longitudinal cohort studies: the MCS and z‐proso. Both studies followed children over an extended period, providing a unique opportunity to examine the developmental trajectories of ED, anger, ADHD symptoms, and parenting practices across two cross‐national contexts.

### Ethics

Study 1 and Study 2 were pre‐registered publicly on the Open Science Framework (OSF) with research questions, hypotheses, and analysis plans: see https://doi.org/10.17605/OSF.IO/V4Z8Q and https://doi.org/10.17605/OSF.IO/NECMD. Ethical approval and informed consent for this paper was obtained from the School of Education Research Committee, Durham University, on 09.05.2024 (EDU‐2024‐05‐09T17_27_19). For the MCS, ethical approval was granted by the NHS Multi‐Centre Research Ethics Committee, with informed parental consent required for child participation (Connelly & Platt, 2014; Hansen et al., 2014; Johnson et al., 2015; Plewis et al., 2007). For z‐proso, ethical approval was granted by the Ethics Committee of the Faculty of Arts and Social Sciences of the University of Zurich (approval numbers: 2018.2.12 and 21.12.13), with active parental consent required until age 15 (Ribeaud et al., 2022; Ribeaud & Gloor, 2023).

### Participants

#### UK MCS

The MCS is a longitudinal study of a representative sample of children born into 19,244 families in the United Kingdom (Plewis et al., 2007). A stratified design and weights provided by the MCS were used to ensure adequate representation of disadvantaged and ethnic minority children and to handle non‐random attrition (Heeringa et al., 2017; Johnson et al., 2015). Fuller descriptions of the design and procedure can be found in Hansen et al. (2014) and Plewis et al. (2007). For the present study, parent and child‐reported data were collected when children were ages 5, 7 and 11 (*n* = 3147–30,135).

#### The Zurich Project on Social Development from Childhood to Adulthood

z‐proso is an ongoing longitudinal study tracking 1675 children who entered public primary school in the city of Zurich in 2004 from age 7 to 24, with data collected across 9 waves. The initial study design was a randomised controlled trial, where 14 quadruplets of schools (56 in total) were randomly selected, stratified by size (number of first graders) and school district. The study is representative of the City of Zurich rather than Switzerland as a whole, reflecting the cultural heterogeneity of this urban sample. Fuller descriptions of the design can be found in Ribeaud et al. (2022). For this study, parent and child‐reported data were collected when children were ages 7, 9, and 11 (*n* = 1179–1360).

### Measures

Descriptive statistics for all scales and waves are provided for both MCS and z‐proso in Tables 1 and 2. In both studies, a range of covariates were used including sex (assigned at birth), socio‐economic status (SES) (z‐proso), income (MCS), maternal education (MCS), children's ethnicity (MCS), and parents' migration background (z‐proso).

**TABLE 1:** Descriptive statistics for MCS.

Variable	*N*	*M*	SD	Min	Max	Range
CSBQ ED (age 5)	14,978	1.36	0.49	5	15	10
SDQ ADHD (age 5)	14,863	3.20	2.37	0	10	10
SCTS parenting—Withdrawn (age 5)	13,831	2.72	1.04	1	6	5
SCTS parenting—Harsh (age 5)	13,831	2.80	0.88	1	6	5
CSBQ ED (age 7)	13,672	1.73	0.47	5	15	10
SDQ ADHD (age 7)	13,605	3.37	2.53	0	10	10
SCTS parenting—Withdrawn (age 7)	13,350	2.59	1.01	1	6	5
SCTS parenting—Harsh (age 7)	13,350	2.72	0.86	1	6	5
SDQ peer problems (age 11)	12,858	1.38	1.70	0	10	10

Abbreviations: ADHD, attention‐deficit/hyperactivity disorder; CSBQ, Child Social Behaviour Questionnaire; ED, emotion dysregulation; SCTS, Straus' Conflict Tactics Scale; SDQ, Strengths & Difficulties Questionnaire.

**TABLE 2:** Descriptive statistics for z‐proso.

Variable	*N*	*M*	SD	Min	Max	Range
SBQ ADHD (age 7—Parent)	1675	23.84	19.80	1	70	69
SBQ ADHD (age 9—Parent)	1675	18.76	15.13	1	55	54
Positive parenting mean (age 9)	1180	3.24	0.34	3.25	0.37	2.88
Negative parenting mean (age 9)	1180	0.94	0.44	0.45	0.93	0.48
Negative parenting mean (age 11)	1072	0.92	0.44	0.43	0.91	0.48
Anger mean dummy‐coded (age 7)	1675	0.27	0.24	0.25	0.25	0
Anger mean dummy‐coded (age 9)	1675	0.29	0.29	0.26	0.37	0.11
Aggressive conflict coping (age 11)	1675	3.52	3.19	1	19	18
Competent conflict coping (age 11)	1675	10.16	7.50	1	24	23

Abbreviations: ADHD, attention‐deficit/hyperactivity disorder; SBQ, Social Behaviour Questionnaire.

#### MCS

##### Child Social Behaviour Questionnaire

ED was measured using the *Emotional Dysregulation subscale* of the Child Social Behaviour Questionnaire (CSBQ; Hartman et al., 2006), which includes five items (see Johnson et al., 2015). Parents rated their child's behaviour over the past 6 months on a 3‐point scale (‘Not true’, ‘Somewhat true’, ‘Certainly true’). The psychometric properties of the CSBQ have been investigated in previous studies, demonstrating its reliability, structural validity, and criterion validity (Hartman et al., 2006). In this sample, omega reliability was good at 0.80 for both ages 5 and 7.

##### Strengths and Difficulties Questionnaire

ADHD symptoms were measured using the Strengths and Difficulties Questionnaire (*SDQ Hyperactivity/Inattention subscale*; Goodman, 1997, 2001), including five items on ADHD‐related behaviours (see Johnson et al., 2015). Responses were rated on a three‐point scale from ‘Not true’ to ‘Certainly true’. Peer relationship problems at age 11 were assessed using the *SDQ Peer Relationship Problems subscale*, including five items on social difficulties. The SDQ's reliability and validity have been well established (Kersten et al., 2016). Omega reliability was 0.75 for the Hyperactivity/Inattention subscale at ages 5 and 7, and 0.71 for the Peer Relationship Problems subscale at age 11.

##### Discipline parenting SCTS

Discipline parenting practices at ages 5 and 7 were measured using the Straus' Conflict Tactics Scale (SCTS; Straus & Hamby, 1997), which includes six items categorised as ‘harsh parenting tactics’ or ‘withdrawn parenting tactics’ (Johnson et al., 2015). Responses were recorded on a five‐point Likert scale. The item ‘bribing with treats’ was excluded from analyses, as it did not align with either category, consistent with prior MCS research (Rajyaguru et al., 2019). The SCTS has strong validity (Straus et al., 1998), as well as reliability, with Omega scores of 0.71 (harsh) and 0.63 (withdrawn) at age 5, and 0.74 (harsh) and 0.67 (withdrawn) at age 7.

#### z‐proso

##### Social Behaviour Questionnaire

ADHD symptoms were measured using an adapted version of the Social Behaviour Questionnaire (SBQ) at ages 7 and 9 (Tremblay et al., 1991; z‐proso Project Team, 2024b). In our study, we focus on parent‐reported data of children's hyperactivity and attention‐deficit symptoms. The SBQ has demonstrated good psychometric properties, including internal consistency and validity (Murray et al., 2019). The average omega score for ADHD symptoms across ages 7 and 9 was 0.62.

##### Social Problem‐Solving Questionnaire

Anger was measured at ages 7 and 9 using the Social Problem‐Solving Instrument based on situational vignettes (Crick & Dodge, 1996; Dodge & Coie, 1987) and adapted by the z‐proso study team (z‐proso Project Team, 2024b). The subdimensions measured in the scale included: (1) 1 question pertaining to likely anger in potentially conflictive situations and (2) up to four repeated questions on number and type of accessible behavioural responses in potentially conflictive situations (Ribeaud & Gloor, 2023). Children were asked to choose one (emotionally arousing) feeling from a choice of: ‘happy’, ‘sad’, ‘angry’, ‘fear’, and ‘no feeling’. For the purposes of this study, we focused on anger, which was dummy coded as 1, with all other feelings being coded as 0 (i.e., happiness, sadness, fear, no feelings). At age 9, four out of the six initial vignettes from Age 7 were used a second time. We focused on anger in the present study (see Background for rationale), and the average omega score for anger at ages 7 and 9 was 0.62.

##### Alabama Parenting Questionnaire

Parenting practices were measured using the Alabama Parenting Questionnaire (APQ; Kliem et al., 2019; Shelton et al., 1996) and the Parenting Scale from the Kriminologisches Forschungsinstitut Niedersachsen (KFN), adapted by the z‐proso Project Team (z‐proso Project Team, 2024b). Responses for both parenting style and sanctioning style were coded on a four‐point Likert scale, ranging from ‘never’ to ‘often/always’ (z‐proso Project Team, 2024b). We grouped the responses into positive (i.e., involvement, positive parenting, control/supervision, and supportive) and negative parenting styles (i.e., erratic parenting, corporal punishment, and aversive parenting), using mean scores per group for analysis. On average, omega scores for positive and negative parenting at Age 9 were 0.62.

##### Conflict Coping Strategies Scale

Conflict coping strategies at age 11 were measured using a shortened version of the KFN scale, adapted by z‐proso. The subdimensions in this scale are divided into two categories: ‘social competent strategy’ or ‘aggressive strategy’ with eight items being measured (z‐proso Project Team, 2024a). Responses were recorded on a five‐point Likert scale from ‘never’ to ‘very often’. On average, omega scores for Age 11 coping strategies (competent and aggressive) were 0.63.

### Statistical procedure

This study uses multiple linear regression (MLR) to examine the effects of gender, SES, maternal education, and ethnicity on ED for the MCS, as well as gender, income, migration background on anger for z‐proso (Field, 2013). Structural equation modelling (SEM) assessed indirect pathways and interactions using R 4.4.1 (Thulin, 2024) and full information maximum likelihood (FIML) estimation for missing data (Enders, 2001), with model fit evaluated using TLI, CFI, and RMSEA (Kline, 2005).

In the MCS sample, we ran cross‐sectional moderation models to examine whether ADHD symptoms moderated the association between withdrawn/harsh parenting practices and emotion dysregulation, at ages 5 and 7, respectively **(H1)**. The MCS sample also examined whether ADHD symptoms at age 7 moderated the association between withdrawn/harsh parenting practices at age 5 and peer relationship problems at age 11, longitudinally **(H2)**. In the z‐proso sample, we ran moderation models to examine whether ADHD symptoms moderated the association between negative/positive parenting and anger, at age 9 **(H3)**. The z‐proso sample additionally focused on whether anger at 9 mediates the association between age 7 negative/positive parenting and conflict coping strategies (aggressive/competent) at age 11, longitudinally **(H4)**.

Statistical assumptions for regression and moderation models, including normality of residuals, linearity, homoscedasticity, and multicollinearity, were assessed using standard diagnostic plots and variance inflation factors (VIF). No significant violations were observed, supporting the validity of the model estimates. All analyses were conducted in R (version 4.3.0) using the lavaan package (version 0.67; Rosseel, 2012) for SEM.

Unstandardised coefficients are presented for models using MCS data to accommodate survey design features and weighting procedures (Pek & Flora, 2018). Standardised estimates for MCS and z‐proso data are provided in Tables S1–S5. R code for MCS and z‐proso data analyses are available on OSF here. Income was measured using OECD equivalised income quintiles (MCS; Horsfield, 2015), SES with the ISEI (z‐proso, Ganzeboom et al., 1992), Maternal education was treated as an ordinal variable based on the UK National Qualification Framework, ranging from no formal qualifications to postgraduate degrees. Children's ethnicity was coded as a nominal categorical variable in line with UK Census classifications: White, Mixed, Asian (Pakistani/Bangladeshi), Black, and Other.

## RESULTS

### MCS findings

H 1 Cross‐sectionally, ADHD symptoms are a key moderator of the relation between withdrawn/harsh parenting practices and ED symptoms, at ages 5 and 7, respectively.

#### Age 5 withdrawn parenting

A moderation model examined the relationship between withdrawn parenting tactics and ED at age 5, with ADHD symptoms as a moderator and covariates including gender, income, maternal education, and ethnicity (*n* = 30,135). The interaction between withdrawn parenting and ADHD symptoms at age 5 was statistically non‐significant (*B* = −0.002, *p* = .072, 95% CI [−0.004, 0.001]), indicating no clear evidence that ADHD symptoms moderated the association between withdrawn parenting and emotion dysregulation. Withdrawn parenting practices showed a statistically non‐significant main effect on emotion dysregulation (*B* = −0.003, *p* = .236, 95% CI [−0.007, 0.001]). The main effect of ADHD symptoms on emotion dysregulation was statistically significant and positive (*B* = 0.106, *p* < .001, 95% CI [0.100, 0.112]). Gender at age 5 had a statistically, non‐significant association with emotion dysregulation (*B* = −0.002, *p* = .725, 95% CI [−0.012, 0.008]). Income showed a statistically non‐significant relationship with emotion dysregulation (*B* = 0.003, *p* = .085, 95% CI [−0.001, 0.007]), as did ethnicity (*B* = −0.002, *p* = .383, 95% CI [−0.006, 0.002]). Higher maternal education was associated with a statistically significant positive effect on emotion dysregulation (*B* = 0.001, *p* < .001, 95% CI [0.001, 0.002]) compared to lower maternal education. This model fit indicated a fully saturated model (CFI = 1.000, TLI = 1.000, RMSEA = 0.000) and explained 18.3% of the variance.

#### Age 5 harsh parenting

A cross‐sectional moderation model (*n* = 30,135) was run for age 5 harsh parenting practices. The interaction between harsh parenting and ADHD symptoms at age 5 was statistically non‐significant (*B* = −0.001, *p* = .747, 95% CI [−0.002, 0.001]), indicating no evidence that ADHD symptoms moderated the association between harsh parenting and emotion dysregulation. The main effect of harsh parenting on emotion dysregulation was also non‐significant (*B* = −0.002, *p* = .349, 95% CI [−0.006, 0.003]). In contrast, the main effect of ADHD symptoms on emotion dysregulation was statistically significant and positive (*B* = 0.102, *p* < .001, 95% CI [0.096, 0.109]). Gender at age 5 had a statistically non‐significant relationship with emotion dysregulation (*B* = −0.002, *p* = .740, 95% CI = [−0.012, 0.008]). Income at age 5 had a statistically non‐significant effect (*B* = 0.003, *p* = .085, 95% CI [−0.0004, 0.0064]), as did children's ethnicity (*B* = −0.002, *p* = .394, 95% CI [−0.0066, 0.0026]) on emotion dysregulation. Higher maternal education at age 5 had a statistically significant positive effect on emotion dysregulation (*B* = 0.001, *p* < .001, 95% CI [0.001, 0.001]), compared to lower maternal education. This model was fully saturated (CFI = 1.000, TLI = 1.000, RMSEA = 0.000), explaining 18.1% of the variance.

#### Age 7 withdrawn parenting

**FIGURE 3:**
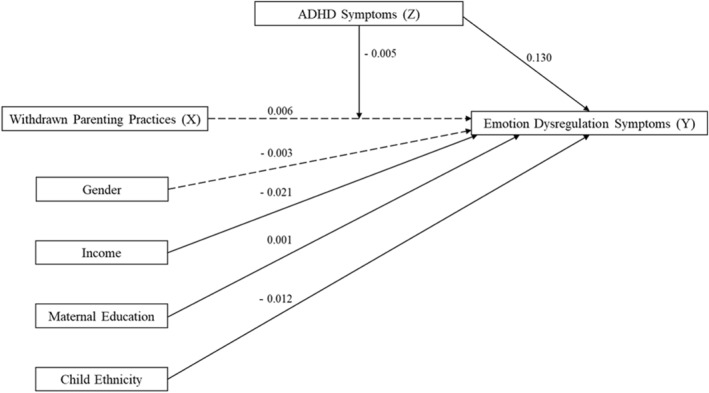
Unstandardised estimates for age 7 moderation: withdrawn parenting, ADHD, and emotion dysregulation (MCS). ADHD, attention‐deficit/hyperactivity disorder; MCS, Millennium Cohort Study.

**FIGURE 4:**
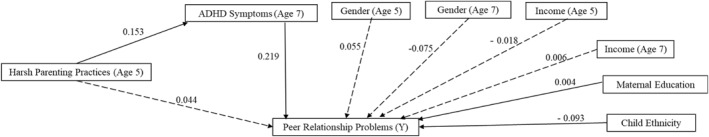
Unstandardised estimates for longitudinal moderation: harsh tactics, ADHD, and peer relationship problems (MCS). ADHD, attention‐deficit/hyperactivity disorder; MCS, Millennium Cohort Study.

**FIGURE 5:**
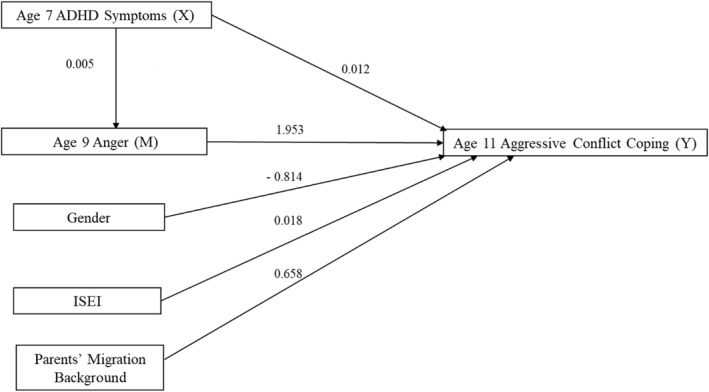
Unstandardised estimates for longitudinal mediation: ADHD, anger, and aggressive coping (ages 7, 9, 11; z‐proso). ADHD, attention‐deficit/hyperactivity disorder; z‐proso, Zurich Project on Social Development from Childhood to Adulthood.

At age 7, the moderation model (*n* = 3147) examined whether ADHD symptoms moderated the association between withdrawn parenting and ED. Unstandardised parameter estimates are presented in Figure 3. Non‐significant associations are marked by dashed lines for Figures 3, 4, 5. ADHD symptoms were positively associated with ED (*B* = 0.130, *p* < .001, 95% CI [0.117, 0.144]), while withdrawn parenting showed no significant main effect (*B* = 0.006, *p* = .360, 95% CI [−0.006, 0.019]). However, a statistically significant negative interaction between ADHD symptoms and withdrawn parenting (*B* = −0.005, *p* = .034, 95% CI [−0.010, −0.001]) indicated that the association between withdrawn parenting and ED was stronger at lower levels of ADHD symptoms. The model explained 13.2% of the variance in ED and, the fit indices indicated a fully saturated model (CFI = 1.000, TLI = 1.000, RMSEA = 0.000).

#### Age 7 harsh parenting

For the age 7 cross‐sectional moderation model of harsh parenting (*n* = 3147), the interaction with ADHD symptoms was not statistically significant (*B* = 0.001, *p* = .720, 95% CI [−0.002, 0.004]), indicating that ADHD symptom levels did not meaningfully moderate the relationship between harsh parenting and emotion dysregulation. The main effect of harsh parenting practices on emotion dysregulation was statistically non‐significant (*B* = 0.001, *p* = .967, 95% CI [−0.010, 0.010]). The main effect of ADHD symptoms at age 7 was statistically significant and positive (*B* = 0.114, *p* < .001, 95% CI [0.103, 0.124]). Gender had a statistically non‐significant effect (*B* = −0.002, *p* = .908, 95% CI [−0.013, 0.009]), with emotion dysregulation. Ethnicity showed a statistically significant and negative effect with emotion dysregulation (*B* = −0.012, *p* = .039, 95% CI [−0.023, −0.001]), as did income (*B* = −0.020, *p* < .001, 95% CI [−0.030, −0.010]). Higher maternal education had a statistically significant positive association with emotion dysregulation (*B* = 0.001, *p* < .001, 95% CI [−0.001, 0.001]), compared to mothers with lower education. The model was fully saturated (CFI = 1.000, TLI = 1.000, RMSEA = 0.000), explaining approximately 13.2% of the variance.

H 2 Longitudinally, ADHD symptoms at age 7 are a key moderator of the relation between withdrawn/harsh parenting practices at age 5 and peer relationship problems at age 11.

#### Withdrawn parenting

Longitudinally, the moderating effect of ADHD symptoms at age 7 was examined between age 5 withdrawn parenting practices and age 11 peer relationship problems (*n* = 4695). The interaction between age 5 withdrawn parenting tactics and age 7 ADHD symptoms showed a statistically significant positive association (*B* = 0.219, *p* < .001, 95% CI [0.199, 0.238]), indicating that the positive association between age 5 harsh parenting and age 11 peer problems is strengthened, as ADHD symptom levels increase along the dimensional spectrum. Withdrawn parenting practices at age 5 showed a statistically non‐significant main effect on peer relationship problems at age 11 (*B* = 0.011, *p* = .63, 95% CI [−0.034, 0.056]). The main effect of ADHD symptoms at age 7 on peer relationship problems at age 11 was statistically significant and positive (*B* = 0.2107, *p* < .001, 95% CI [0.190, 0.231]). Age 5 gender showed a statistically non‐significant association with age 11 peer relationship problems (*B* = 0.055, *p* = .260, 95% CI [−0.043, 0.154]), as did age 7 gender (*B* = −0.074, *p* = .127, 95% CI [−0.168, 0.020]). Income at age 5 was a statistically non‐significant with peer relationship problems (*B* = −0.018, *p* = .297, 95% CI [−0.052, 0.016]), as was income at age 7 (*B* = 0.007, *p* = .704, 95% CI [−0.026, 0.038]). Children's ethnicity showed a statistically significant, negative association with age 11 peer relationship problems (*B* = −0.092, *p* < .001, 95% CI [−0.137, −0.047]). Higher maternal education was statistically significant and positively associated with peer problems, compared to lower levels of maternal education (*B* = 0.004, *p* < .001, 95% CI [0.002, 0.006]). The model fit was fully saturated (CFI = 1.000, TLI = 1.000, RMSEA = 0.000) and the variance explained by the model was 18.9%.

#### Harsh parenting

Longitudinally, the moderating effect of ADHD symptoms at age 7 on the relationship between age 5 harsh parenting practices and age 11 peer relationship problems was examined (*n* = 4695). Unstandardised parameter estimates for this model are presented in Figure 4. The interaction between harsh parenting and ADHD symptoms was statistically significant, with a positive association (*B* = 0.219, *p* < .001, 95% CI [0.199, 0.238]), indicating that the association between harsh parenting at age 5 and peer relationship problems at age 11 becomes stronger as ADHD symptom levels increase. The main effect of harsh parenting at age 5 showed a non‐significant positive association with age 11 peer problems at age 11 (*B* = 0.044, *p* = .107, 95% CI [−0.008, 0.097]). The main effect of ADHD symptoms at age 7 was also statistically significant, showing a positive association with peer problems at age 11 (*B* = 0.153, *p* < .001, 95% CI [0.138, 0.168]). For gender, a statistically non‐significant association was found at age 5 (*B* = 0.055, *p* = .260, 95% CI [−0.043, 0.154]), as well as age 7 (*B* = −0.075, *p* = .122, 95% CI [−0.169, 0.019]). Income at age 5 had a statistically non‐significant association with peer relationship problems (*B* = −0.018, *p* = .298, 95% CI [−0.051, 0.015]), as did income at age 7 (*B* = 0.006, *p* = .73, 95% CI [−0.027, 0.039]). Ethnicity was statistically significant and showed a negative association with age 11 peer relationship problems (*B* = −0.093, *p* < .001, 95% CI [−0.136, −0.051]). Higher maternal education was statistically significant, showing a positive association with peer problems (*B* = 0.004, *p* < .001, 95% CI [0.003, 0.005]), compared to lower levels of maternal education. The model was fully saturated (CFI = 1.000, TLI = 1.000, RMSEA = 0.000) and explained 18.9% variance.

### z‐proso findings

H 3 Cross‐sectionally, ADHD symptoms are a key moderator of the relation between negative/positive parenting practices and anger, at age 9.

#### Age 9 negative parenting

Cross‐sectionally, we examined if ADHD symptoms at age 9 moderated the relationship between negative parenting and anger, controlling for gender, SES, and migration status (*n* = 1179). Negative parenting had no significant main effect on anger (*B* = −0.002, *p* = .903, 95% CI [−0.041, 0.036]), and ADHD symptoms did not predict anger (*B* = 0.007, *p* = .614, 95% CI [−0.018, 0.032]). The interaction between ADHD and negative parenting was statistically non‐significant (*B* = −0.024, *p* = .354, 95% CI [−0.074, 0.026]), indicating that ADHD symptom levels did not meaningfully moderate the relationship between negative parenting and anger symptoms. The model explained 14% of the variance in anger and was fully saturated (CFI = 1.000, TLI = 1.000, RMSEA = 0.000).

#### Age 9 positive parenting

Cross‐sectionally, we also examined if ADHD symptoms at age 9 moderated the relationship between positive parenting and anger, controlling for gender, SES, and migration status (*n* = 1179). Positive parenting did not significantly predict anger (*B* = −0.027, *p* = .278, 95% CI [−0.074, 0.020]), nor did ADHD symptoms (*B* = 0.004, *p* = .728, 95% CI [−0.020, 0.028]). The interaction between positive parenting and ADHD symptoms was statistically non‐significant (*B* = −0.011, *p* = .742, 95% CI [−0.078, 0.056]), indicating that ADHD symptom levels did not meaningfully moderate the relationship between positive parenting and anger symptoms. The model explained 14% of the variance in anger and was fully saturated (CFI = 1.000, TLI = 1.000, RMSEA = 0.000).

H 4 Longitudinally, anger at age 9 is a key mediator of the relation between ADHD symptoms at age 7 and conflict coping strategies at age 11 in childhood.

A longitudinal mediation model (*n* = 1180) tested age 9 anger as a mediator between ADHD symptoms at 7 and aggressive conflict coping at 11, controlling for gender, SES, and migration background. Unstandardised parameter estimates are provided in Figure 5. ADHD symptoms at age 7 influenced anger at age 9 (*B* = 0.003, *p* < .001, 95% CI [0.002, 0.004]) and coping at age 11, both directly (*B* = 0.012, *p* = .012, 95% CI [0.003, 0.021]) and indirectly via anger (*B* = 0.005, *p* < .001, 95% CI [0.003, 0.007]). Anger strongly predicted aggressive coping (*B* = 1.953, *p* < .001, 95% CI [1.350, 2.556]). The total effect was significant (*B* = 0.018, *p* < .001, 95% CI [0.008, 0.028]). For this model, ADHD symptoms at age 7 had a small, statistically significant direct effect on anger (*B* = 0.003, *p* < .001, 95% CI [0.002, 0.004]), with more pronounced ADHD symptoms linked to greater anger at age 9. Anger strongly predicted competent coping at age 11 (*B* = 4.928, *p* < .001, 95% CI [3.705, 6.151]). ADHD symptoms indirectly affected competent coping via anger (*B* = 0.013, *p* < .001) and had a modest total effect (*B* = 0.032, *p* = .002). The model explained 7% variance in anger and was fully saturated (CFI = 1.000, RMSEA = 0.000).

### Sensitivity analyses

Recent research suggests that internalising difficulties in middle childhood, such as anxiety and sadness, may precede externalising behaviours through impaired emotion regulation, highlighting the importance of understanding how negative emotions shape coping responses (Keskin et al., 2023). To explore this, a sensitivity analysis was conducted using a latent variable model of negative emotions (sadness, anger, and fear) predicting two coping dimensions: competent conflict coping and aggressive conflict coping with both models having a sample of *n* = 1309 (see p. 86 in the z‐proso R code here). Results indicated small, statistically non‐significant associations with competent coping (*B* = 0.183, *p* = .735, 95% CI [0.098, 0.267]) and aggressive coping (*B* = 0.196, *p* = .473, 95% CI [0.082, 0.310]), with acceptable model fit (CFI = 0.815, RMSEA = 0.068). These findings may reflect the complexity of emotion‐coping associations in middle childhood, potentially moderated by unmeasured factors such as individual differences in self‐regulation or contextual influences like parenting and peer dynamics (Graziano & Garcia, 2016). As such, future work should investigate these interacting influences to better understand the pathways linking early emotional difficulties to behavioural responses in children with ADHD.

## DISCUSSION

This study explored how ADHD symptoms and anger interact with parenting practices to influence ED, peer relationship problems, and conflict coping strategies, drawing on longitudinal data from the UK MCS and the Swiss z‐proso cohort. Our findings partially supported the hypotheses, revealing complex pathways linking early ADHD symptoms, parenting styles, anger, and social outcomes across cross‐national contexts. Notably, ADHD symptoms predicted greater ED and anger, with withdrawn parenting exacerbating emotional difficulties in children, while anger mediated the relationship between ADHD symptoms and conflict coping strategies.

### MCS

Cross‐sectionally, ADHD symptoms predicted ED at age 5, with withdrawn parenting strengthening this association. Interestingly, by age 7, withdrawn parenting's effect on ED was weaker among children with higher ADHD symptoms, a finding that contrasts with prior research suggesting that children with more severe ADHD symptoms are more vulnerable to negative parenting (Breaux et al., 2018). One explanation is a potential ceiling effect: children with higher ADHD symptoms may already experience elevated baseline ED, making additional parenting effects less pronounced. Conversely, children with lower ADHD symptoms might be more sensitive to variations in parenting style, showing greater increases in ED when exposed to withdrawn parenting. Longitudinally, ADHD symptoms at age 7 intensified the negative impact of both harsh and withdrawn parenting at age 5 on peer difficulties at age 11, consistent with prior studies highlighting the combined risk of ADHD symptoms and adverse parenting for social maladjustment (Berthelon et al., 2020; Bussing et al., 2011).

### z‐proso

Cross‐sectionally at age 9, ADHD symptoms robustly predicted anger, supporting literature on heightened emotional reactivity and frustration intolerance in ADHD populations (Evans et al., 2014, 2017; Lee et al., 2021). Longitudinally, anger at age 9 partially mediated the association between ADHD symptoms at age 7 and conflict coping at age 11. This supports the dual pathway model of ADHD (Sonuga‐Barke, 2003), which proposes two mechanisms linking ADHD to social and emotional difficulties: executive dysfunction and emotion regulation impairments. Our findings emphasise the latter, with anger dysregulation serving as a critical emotional process connecting ADHD symptoms to maladaptive conflict coping strategies. Interestingly, anger was also associated with competent coping strategies, suggesting that well‐regulated anger may facilitate adaptive social functioning, aligning with emerging views of emotion regulation as a dynamic process rather than solely pathological (Halperin & Schulz, 2006).

### Strengths

A major strength of this study lies in its longitudinal design, which spans three developmental time points across two Western, cross‐national, population‐based cohorts. This structure enabled the investigation of developmental trajectories within and across cultural contexts, offering a rare opportunity to examine both within‐sample change and cross‐national comparability. Such a design significantly enhances the generalisability and ecological validity of the findings. Moreover, the application of advanced statistical techniques, including SEM and FIML estimation, allowed for rigorous handling of missing data and complex model structures, thereby improving the precision, robustness, and interpretability of the results (Enders, 2001).

### Limitations

However, several limitations warrant consideration. Unmeasured factors, such as genetic predispositions, may influence anger and aggression, suggesting that future research incorporating genetic data could provide further insights (Cupaioli et al., 2021). Parent‐reported measures may also introduce bias, and including multiple informants (e.g., teacher reports) would offer a more accurate view (DuPaul et al., 2016). Additionally, we acknowledge the important issue of shared method variance. As all measures in this study were parent/child‐reported, associations among constructs such as ADHD symptoms, parenting practices, anger, and emotion dysregulation may be inflated due to common rater effects. Future research should incorporate multi‐informant and multi‐method approaches (e.g., observational data, physiological measures) to reduce shared method bias and provide a more comprehensive understanding. Moreover, differences in measurement tools between cohorts (e.g., SDQ vs. SBQ) may affect comparability, and standardising instruments would enhance cross‐national consistency (Goodman, 2001).

While the present study examined mediation and moderation processes separately across two cohorts, future research could explore the potential for a moderated mediation model, particularly within the z‐proso sample. Such an approach would allow for testing whether the indirect effect of negative parenting on conflict coping strategies via anger is conditioned by ADHD symptoms. Although this level of complexity was beyond the scope of the current analysis, it represents a promising avenue for further investigation, particularly in studies with sufficient statistical power and longitudinal depth to clarify developmental timing and causal pathways.

### Clinical implications

The intricate relationships between ADHD symptoms, parenting, anger, and social outcomes underline the importance of early and targeted interventions. Recent psychosocial interventions explicitly addressing ED and parenting in children with ADHD have shown promise. For instance, the RELAX intervention (Breaux & Langberg, 2020) targets emotion dysregulation and interpersonal conflict in adolescents with ADHD, demonstrating improvements in emotional and social functioning. Similarly, the Managing Frustration for Children (MFC) program (Rosen et al., 2019) offers a group‐based approach to enhance emotion regulation skills among children with ADHD, reducing frustration and aggression. A systematic review by Vacher et al. (2020) further highlights the efficacy of psychosocial interventions focusing on ED in ADHD populations, emphasising the need for integrated approaches that address both child emotional processes and parenting practices. Our findings reinforce these clinical insights, suggesting that interventions combining parenting support with emotion regulation training during middle childhood could be critical for preventing long‐term social difficulties in children with ADHD.

Future studies should explore how gender, culture, and socio‐economic factors moderate the interplay between ADHD symptoms, parenting, ED, and social outcomes. Longitudinal RCTs testing integrated interventions targeting both parenting and child emotion regulation would clarify causal pathways and identify effective treatment components. Additionally, standardising measurement tools across diverse populations will enhance cross‐cultural validity and intervention tailoring.

## CONCLUSION

This study advances understanding of how ADHD symptoms, anger, and parenting interact over time to shape emotional and social development in children across two distinct cultural settings. Our findings highlight the critical role of emotion regulation, particularly anger management, in mediating the relationship between ADHD symptoms and social outcomes. Importantly, they underscore the necessity of culturally sensitive, developmentally timed interventions focusing on both parenting practices and child emotion dysregulation to improve peer relationships and conflict coping in children with ADHD, particularly those facing socio‐economic disadvantage.

## AUTHOR CONTRIBUTIONS

**Evelyn Mary‐Ann Antony**: Conceptualisation; funding acquisition; software; data curation; formal analysis; investigation; methodology; project administration; resources; validation; visualisation; writing—original draft preparation; writing—reviewing and editing. **Nadin Beckmann**: Funding acquisition; supervision; writing—reviewing and editing. **Steve Higgins**: Supervision.

## CONFLICT OF INTEREST STATEMENT

The authors declare no conflicts of interest.

## ETHICAL CONSIDERATIONS

Ethical approval and informed consent for this paper was obtained from the School of Education Research Committee, Durham University, on 09.05.2024 (EDU‐2024‐05‐09T17_27_19). For the MCS, ethical approval was granted by the NHS Multi‐Centre Research Ethics Committee, with informed parental consent required for child participation (Connelly & Platt, 2014; Hansen et al., 2014; Johnson et al., 2015; Plewis et al., 2007). For z‐proso, ethical approval was granted by the Ethics Committee of the Faculty of Arts and Social Sciences of the University of Zurich (approval numbers: 2018.2.12 and 21.12.13), with active parental consent required until age 15 (Ribeaud et al., 2022; Ribeaud & Gloor, 2023).

## Supporting information

Tables S1–S5

## Data Availability

For MCS, data is openly available in a public repository at https://ukdataservice.ac.uk/ that does not issue DOIs (Project id: 257618). For z‐proso, data is not available, due to privacy and ethical restrictions.
