# Supplementary material for: A dual cohort analysis of parenting practices, attention‐deficit/hyperactivity disorder symptoms, anger, and emotion dysregulation in middle childhood: Findings from a UK and Zurich sample
Source: JCPP Adv. 2025 Oct 19;6(2):e70059. doi: 10.1002/jcv2.70059 (PMC13260677; doi:10.1002/jcv2.70059)
Supplement: Supplementary file 1 — Tables S1–S5 [file JCV2-6-e70059-s001.docx]

## A Dual Cohort Analysis of Parenting Practices, ADHD Symptoms, Anger, and Emotion Dysregulation in Middle Childhood: Findings from a UK and Zurich Sample

## Supporting Information

## Table S1. Multiple Linear Regression Results for ED and Peer Relationship Problems (MCS)

| Outcome Variable | Predictor | β | *p* |
| --- | --- | --- | --- |
| Age 5 ED | Gender | .067 | < .001 |
|  | Income | -.003 | .883 |
|  | Maternal Education | .002 | < .001 |
|  | Child Ethnicity | -.005 | .260 |
| Age 7 ED | Gender | -.010 | .642 |
|  | Income | -.010 | .190 |
|  | Maternal Education | .002 | < .001 |
|  | Child Ethnicity | -.007 | .491 |
| Age 11 Peer Relationship Problems | Gender  Income  Maternal Education  Child Ethnicity | -.042  -.003  .006  -.049 | .259  .822  <.001  .004 |
|  |  |  |  |
|  |  |  |  |

## Table S2. Multiple Linear Regression Results for Anger (z-proso)

| Predictor | β | *p* |
| --- | --- | --- |
| Gender | -1.065 | < .001 |
| ISEI | 4.113 | .054 |
| ISEI  ISEI | 1.261  3.114 | .041  .041 |
|  |  |  |
| Migration Status (Parent Born in Switzerland) | 0.001 | .999 |
| Migration Status (Migrant Parents) | -0.466 | .208 |

## Table S3. Moderation Model Results: Parenting Tactics and ADHD Symptoms (MCS)

| Parenting Practice and Interaction | β | *p* |
| --- | --- | --- |
| Withdrawn Parenting (Age 5) | -.003 | .236 |
| ADHD Symptoms (Age 5) | .106 | < .001 |
| Withdrawn Parenting × ADHD (Age 5) | -.002 | .072 |
| Harsh Parenting (Age 5) | -.002 | .349 |
| ADHD Symptoms (Age 5) | .102 | < .001 |
| Harsh Parenting × ADHD (Age 5) | -.001 | .747 |
| Withdrawn Parenting (Age 7) | .006 | .360 |
| ADHD Symptoms (Age 7) | .130 | < .001 |
| Withdrawn Parenting × ADHD (Age 7) | -.005 | .034 |
| Harsh Parenting (Age 7) | .000 | .967 |
| ADHD Symptoms (Age 7) | .114 | < .001 |
| Harsh Parenting × ADHD (Age 7) | .001 | .720 |

## Table S4. Moderation Model Results: Negative and Positive Parenting (z-proso)

| Parenting Practice and Interaction | β | *p* |
| --- | --- | --- |
| Negative Parenting (Age 9) | -.002 | .903 |
| ADHD Symptoms (Age 9) | .007 | .614 |
| Negative Parenting × ADHD (Age 9) | -.024 | .354 |
| Positive Parenting (Age 9) | -.027 | .278 |
| ADHD Symptoms (Age 9) | .004 | .728 |
| Positive Parenting × ADHD (Age 9) | -.011 | .742 |

## Table S5: Mediation Model Results: ADHD, Anger, and Conflict Coping (z-proso)

| Path | β | *p* |
| --- | --- | --- |
| ADHD (Age 7) → Anger (Age 9) | .003 | < .001 |
| Anger (Age 9) → Aggressive Coping (Age 11) | 1.953 | < .001 |
| Indirect Effect (ADHD → Anger → Aggressive Coping) | .005 | < .001 |
| ADHD (Age 7) → Anger (Age 9) | .003 | < .001 |
| Anger (Age 9) → Competent Coping (Age 11) | 4.928 | < .001 |
| Indirect Effect (ADHD → Anger → Competent Coping) | .013 | < .001 |
